# Construction and characterization of EGFP reporter plasmid harboring putative human *RAX* promoter for in vitro monitoring of retinal progenitor cells identity

**DOI:** 10.1186/s12860-021-00378-2

**Published:** 2021-08-04

**Authors:** Atefeh Atefi, Pendar Shojaei Kojouri, Fereshteh Karamali, Shiva Irani, Mohammad Hossein Nasr-Esfahani

**Affiliations:** 1 Department of Biology, Science and Research Branch, Islamic Azad University, Tehran, Iran; 2 Department of Animal Biotechnology, Cell Science Research Center, Royan Institute for Biotechnology, ACECR, Isfahan, Iran

**Keywords:** Human retinal progenitor cells, Retinal regeneration, Proliferation capacity, *RAX* promoter

## Abstract

**Background:**

In retinal degenerative disease, progressive and debilitating conditions result in deterioration of retinal cells and visual loss. In human, retina lacks the inherent capacity for regeneration. Therefore, regeneration of retinal layer from human retinal progenitor cells (hRPCs) is a challenging task and restricted in vitro maintenance of hRPCs remains as the main hurdle. Retina and anterior neural fold homeobox gene (*RAX*) play critical roles in developing retina and maintenance of hRPCs. In this study, for the first time regulatory regions of human *RAX* gene with potential promoter activity were experimentally investigated.

**Results:**

For this purpose, after in silico analysis of regulatory regions of human *RAX* gene, the expression of EGFP reporter derived by putative promoter sequences was first evaluated in 293 T cells and then in hRPCS derived from human embryonic stem cells. The candidate region (*RAX*-3258 bp) showed the highest EGFP expression in hRPCs. This reporter construct can be used for in vitro monitoring of hRPC identity and verification of an efficient culture medium for maintenance of these cells.

**Conclusions:**

Furthermore, our findings provide a platform for better insight into regulatory regions of human *RAX* gene and molecular mechanisms underlying its vital functions in retina development.

## Background

Retinitis pigmentosa (RP) and age-related macular degeneration (AMD) are the most common types of retinal degeneration disease (RDD) [1]. In RDD, retinal cells are damaged and thereby visual ability is impaired [2]. The intrinsic regenerative capacity of the human retina is extremely restricted and there is a growing focus on using human retinal progenitor cells (hRPCs) as a potential therapeutic approach for restoring retinal function and visual ability [3]. In this regard, RPCs can reduce disease progression rate by secretion of growth factors and upon integration and differentiate into new rod and cone photoreceptors in retinal layer, can facilitate the process of visual rehabilitation [4–6]. In addition, transplanted RPCs exhibit low tumorigenic potential and have tendency to differentiate into retinal cell layers [7]. However, their restricted in vitro maintenance and proliferation capacity have hampered their application in the field of regenerative medicine. It has been shown that they lost their proliferative ability after maximum of seven passages, with reduced capacity for retinal cells formation [3]. One indirect approach to assess hRPCs preservation while being maintained in vitro, is to target their cell morphology and molecular based tracking such as immunostaining. However, a more direct alternative is to use the advantage of a fluorescent reporter under control of a specific retinal promoter [8]. Retina and anterior neural fold homeobox gene (*RAX*) is one of the initial genes expressed in prospective retina derived from the anterior neural plate [9]. RAX transcription factor consists of two conserved domains of homeodomain proteins. The first one is an octapeptide motif in the N-terminal and the second one is a C-terminal OAR (otp, aristaless, and rax) domain, which play prominent roles in the eye development [10]. RAX is essential for the eye field specification and normal development of retina. It is generally down-regulated during differentiation towards retinal cells [11]. Previous researches showed positive correlation between RAX expression and RPCs proliferation in mouse and xenapous [12, 13]. Human *RAX* promoter region has not been extensively examined yet [14]. In this study, after prediction of putative human *RAX* promoter regions, the expression of EGFP reporter derived by these regulatory regions was first evaluated in 293T cells and then in human embryonic stem cell (hESC) derived hRPCs. Distal region of human *RAX* gene containing − 3097 to + 161 resulted in the highest EGFP expression in hRPCs. Altogether, our findings provide a better understanding of regulatory regions of human *RAX* gene, and can be extended to study the mechanism behind RAX function in multipotent retinal progenitors. The identification of human *RAX* promoter sequence might be a valuable supplementary tool for assessment of molecular pathways involved in retinal proliferation and differentiation.

## Results

### Verification of hESCs differentiation into hRPCs

**Fig. 1:**
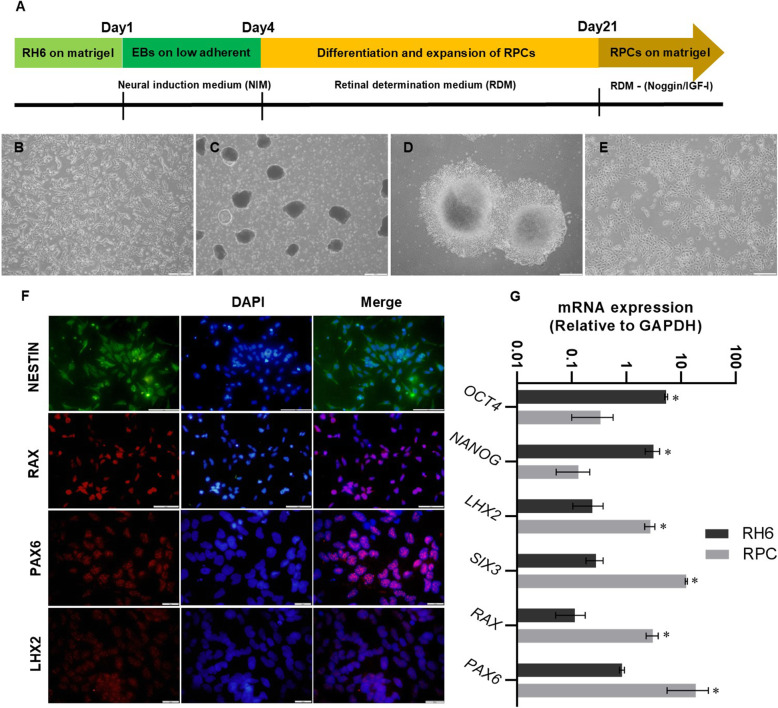
Overview of retinal differentiation and characterization of hPSCs-derived RPCs. **A** Stepwise process of hPSCs differentiation into RPCs. **B** Phase contrast images of hPSCs cultured on Matrigel coated dish, **C** hPSC derived EBs cultured in low adherent dishes, **D** Expansion of RPCs from EBs on Matrigel coated dishes and (**E**) Expansion of hRPCs 21 days after seeding. **F** Immunocytochemistry of eye field-associated transcription factors: RAX, PAX6, LHX2 (red) and NESTIN (green). Scale bars: 100 μm and 50 μm (**G**) qPCR analysis of stemness and EFTF biomarkers in hPSCs and RPCs (*p < 0.05 vs control, *n* = 3)

After differentiation of hESCs to hRPCs by RDM, the identity of hRPCs was confirmed by immunocytochemical analysis of eye filed transcription factors (EFTF) panel including LHX2, RAX, PAX6, SIX3 and also stemness markers like OCT4 and NANOG (Fig. 1F, G). According to Fig. 1, differentiation of hESCs towards retinal progenitors was verified by significant reduction of OCT4 and NANOG, whereas EFTFs had successfully higher protein expression in derived hRPCs. For further confirmation of proper retinal development, the relative expression of EFTFs were analyzed at mRNA level by quantitative RT-PCR. Based on our results, the expression of anterior neural and eye field genes including *SIX3*, *RAX* and *PAX6* were increased when cells cultured in RDM compared to hESCs. Moreover, the expression of stemness markers including *OCT4* and *NANOG*, were significantly decreased after differentiation of ESCs into hRPCs (Fig. 1G). These results successfully confirmed the potential of selected medium for differentiation of human stem cells towards retinal progenitor cells.

### In silico analysis of *RAX* promoter

In this study UCSC genome browser was applied for analysis of chromatin structure (DNase hypersensitivity), and chromatin state of human *RAX* gene upstream region.

**Fig. 2:**
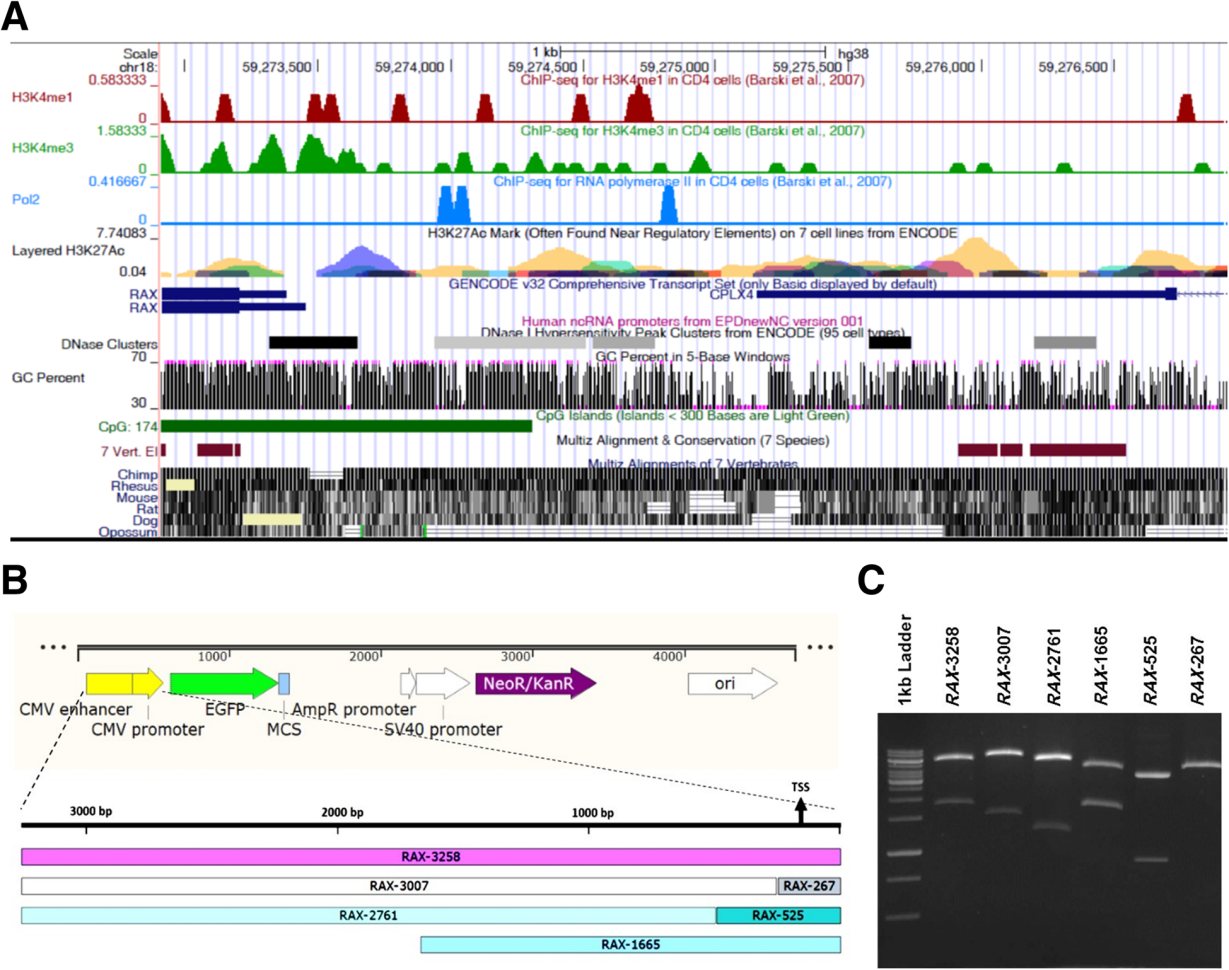
Analysis of putative regulatory regions of human *RAX* gene to derive EGFP reporter plasmids. **A** Screenshot from UCSC genome browser indicating human *RAX* gene upstream region, H3K4me1, H3K4me3, H3K27ac and Pol2 patterns, CpG islands and DNase clusters. **B** Schematic representation of pEGFP-C1 plasmid in which CMV promoter was substituted with putative promoter regions of human *RAX* gene. **C** The final expression plasmids were confirmed by restriction digestion analysis

DNase I hypersensitivity marks different classes of cis regulatory elements within genome, such as promoters and enhancers [15]. UCSC analysis showed several subset of DNase I clusters within ~ 3 kb region upstream of human *RAX* gene (chr18: 59,273,233–59,276,490). Also, Fig. 2A demonstrated the distribution of H3K4me1, H3K4me3 and H3K27ac in 5′ upstream region of *RAX* gene, which are marks of active promoter and enhancer regions.

Based on ChIP-seq analysis in UCSC database, there are three fragments upstream of transcription start site of human *RAX* gene enriched of RNA polymerase II which can be indicator of promoter activity of this region [16].

CpG islands are typically located near TSS and might be associated with promoter regions [17]. In silico analysis of upstream region of *RAX* gene by UCSC genome browser confirmed the presence of a CpG island of around 1 kb length. Moreover, these results showed high evolutionarily conservation of this sequence in primates which reflects the significance of elements contained in this genomic regulatory region. Collectively based on these results 3258 bp upstream of human *RAX* gene was selected as a putative region with promoter activity.

In order to have a better view of potential regulation of *RAX* gene at transcriptional level, distal region was also analyzed for putative binding sites of transcription factors involved in retinal progenitor cell proliferation or development. For this purpose, GTRD which is a collection of ChiP-seq database for identification of TFBS in human and mouse, and also JASPAR data base were analyzed.

**Fig. 3:**
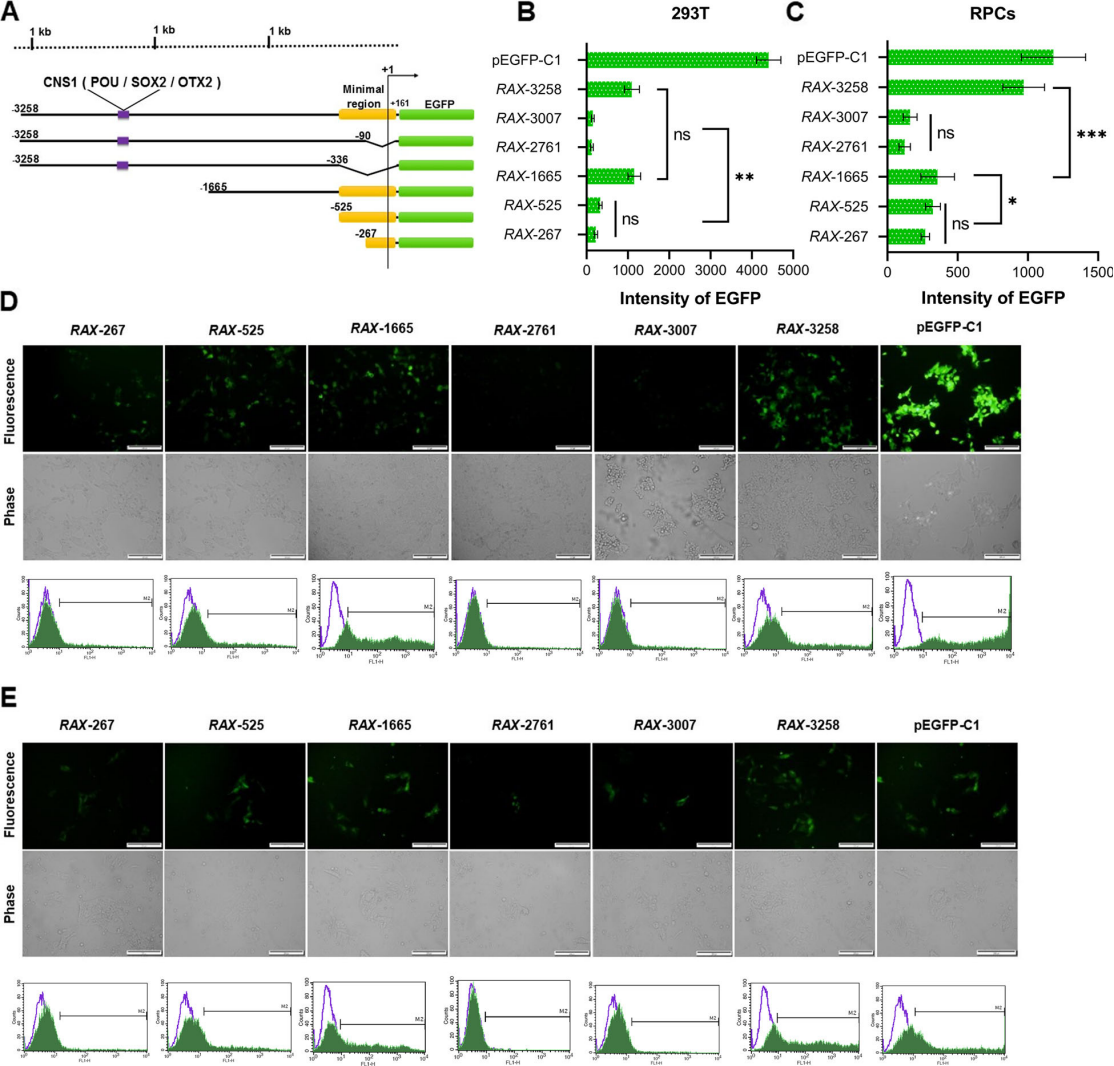
Deletion analysis of human *RAX* promoter regions. **A** A series of 5′ deletions from − 3258 to + 161 bp of human *RAX* gene. **B**, **C** Intensity of EGFP reporter in promoter deletion constructs was determined by flow cytometry in 293 T cells (B) and RPCs (**C**). Data are expressed as mean ± SD (*p < 0.05, ** *p* < 0.005, ***p < 0.0005, *n* = 3). **D**, **E** Comparison of EGFP expression derived by different *RAX* regulatory regions in 293 T cells (**D**) and RPCs (**E**) using flow cytometry and fluorescence microscopic analysis. The scale bar is 100 μm

This sequence analysis revealed the presence of several presumptive binding sites for SOX2 and OTX2 in distal region of human *RAX* gene in accordance with previous studies in Xenopus which was introduced as a conserved noncoding sequence (CNS1) by Danno et.al (Fig. 3A) [18]. UCSC analysis revealed that CNS1 is highly conserved in primates and rodents (Fig. 2A). Also a number of putative binding sites were predicted for SMAD2/3 within human *RAX* gene. SMAD2/3 has been introduced as a key mediator for in vitro differentiation of mouse ESCs into retinal cells by direct binding to the regulatory elements of vital genes like *Rax*, *Pax6 and Otx2* [19].

### Deletion analysis of human *RAX* promoter

**Fig. 4:**
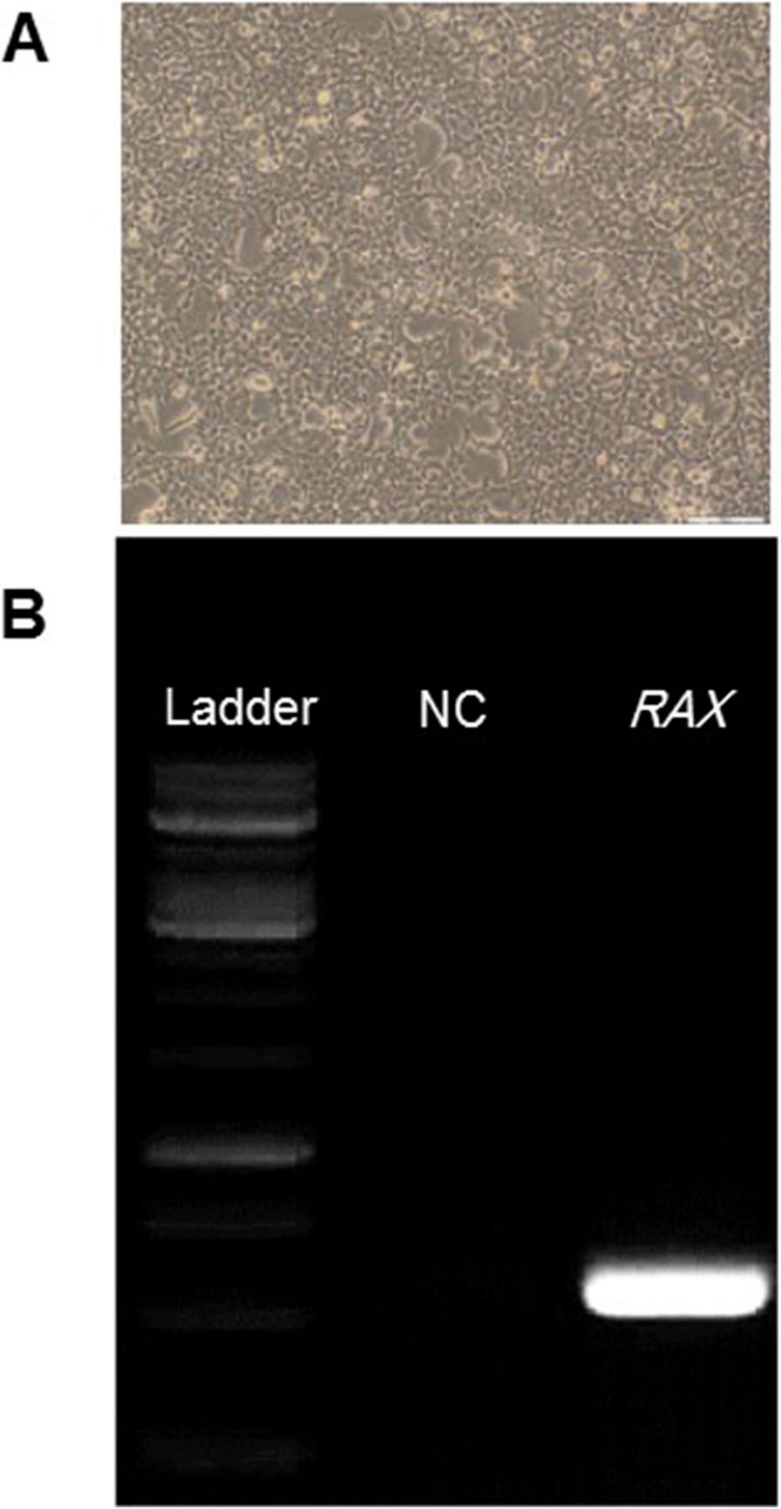
*RAX* expression result was confirmed by PCR amplification. **A** Phase contrast image of 293T cell line. **B** PCR amplification of *RAX* gene (107 bp) with synthesized cDNA from 293T isolated total RNA, 50 bp DNA ladder

To experimentally investigate the promoter activity of 5′ flanking region of human *RAX* gene, we performed EGFP reporter assay using deletion constructs of regulatory regions. For this purpose, different candidate regulatory fragments were cloned upstream of EGFP reporter (Fig. 3A) and transiently transfected in 293T cells (Fig. 3B, D) and hRPCs (Fig. 3C, E). Based on protein atlas database 293 T cells exhibited a low level of *RAX* mRNA expression. RT-PCR results also confirmed the amplification of *RAX* gene (NM_013435) with 107 bp size with synthesized cDNA from 293T total RNA (Fig. 4A, B). So this cell line with high transfection efficiency was selected for analysis of *RAX* promoter activity. Before transfecting putative promoter region into target cells, the integrity of expression constructs was examined by restriction digestions (Fig. 2B, C) and sequencing analysis (data not shown). Based on flow cytometry analysis 48 h post transfection, minimal upstream sequences of *RAX* gene with 267 and 525 bp length resulted in low rate of EGFP expression in 293T cells. After elimination of these two minimal regions, 3007 and 2761 bp fragments indicated lower EGFP expression compared to the minimal regions. On the other hand, 1665 and 3258 bp regions could highly derive the EGFP reporter and with a similar expression rate (Fig. 3B). Surprisingly, 3258 bp distal region indicated the highest *RAX* promoter activity in retinal progenitor cells and contributed to 2-fold EGFP enhancement in compared to 1665 proximal promoter sequence (Fig. 3C). These results suggest that there might be enhancer elements in distal region of human *RAX* gene which might cooperate with minimal regions and thereby modulating transcription of the associated *RAX* gene.

## Discussion

In retina, proliferation, differentiation and cell fate decision are among cellular events being controlled by complex extrinsic and intrinsic signals [20, 21]. Transcription factors such as PAX6, SIX3, OTX2, SOX2 and RAX are intrinsic regulators of maintenance and development of RPCs [22, 23].

In vertebrates, RAX transcription factor plays indispensable roles in early development of retina and has been implicated in RPCs maintenance [12]. Deletion studies of *Rax* in mice led to loss of optic vesicle development [24]. Moreover, studies have been showed that conditional knockout of *Rax* resulted in failure of laminar structure formation in retina, reduction of retinal progenitor cells, and retinal cell fate changes in conditional knockout mouse model [25]. However, there are still ambiguous aspects of RAX transcription factor roles in mammalian eye formation.

Although, most of the retinal disorders are resulted from destruction of different retinal cell types [26], providing a sufficient pool of RPCs for cell therapy as clinical approach is presently unachievable. Since in vitro culture of ESC derived RPCs, leads to down regulation of pivotal EFTFs in these progenitor cells and loss of their identity over passages [27–29], this limitation results in failure of providing adequate pool of RPCs for downstream studies. Therefore, to monitor in vitro maintenance of ESCs-derived hRPCs, an expression vector driving EGFP reporter by human *RAX* promoter was designed. For this purpose, we experimentally investigated the regulatory regions upstream of human *RAX* gene with potential promoter activity. This approach can facilitate further researches in optimization of in vitro culture condition, like analysis of different growth factors and small molecules, required for maintenance of RPCs with high capability of proliferation and multipotency. With providing adequate number of RPCs, animal studies and clinical trials can be more successfully performed in hope of achieving several cell-based therapy approaches for diseases such as AMD and RP, as most common retinal disease.

First we characterized hRPCs derived from hESCs by expression analysis of eye field markers [30]. In our previous studies, Noggin, IWR and IGF-1 were included to the culture medium to differentiation of hESCs into hRPCs [31]. In this protocol, several sequential induction steps are needed to achieve retinal progenitor cells. For development of forebrain derivatives, BMP and Wnt/ß-catenin pathways should be antagonized [32, 33]. Therefore, in order to direct ESCs to the anterior neural fate, EBs were treated with combination of noggin (a potent inhibitor of BMP pathway), IWR (an antagonist of Wnt/ß-catenin signaling pathway) and IGF-1as an inducer of retinal progenitors from ESCs under 3D culture conditions [34].

The prediction of putative promoter region was performed by using different bioinformatics tools. Human *RAX* promoter region has not been thoroughly studied so far. For this purpose, upstream flanking region of human *RAX* gene was analyzed by UCSC in terms of chromatin state (H3K4me1, H3K4me3 and H3K27ac), DNase hypersensitive sites, CpG islands, POL II enrichment and sequence conservation [35]. DNase clusters indicated that transcriptional machinery might be enriched at these particular sequences with open chromatin structure [36]. Furthermore, our in silico analysis demonstrated the distribution of H3K4me1, H3K4me3 and H3K27ac in 5′ upstream region of human *RAX* gene. H3K4me1 and H3K4me3 epigenetic modifications are normally signature of active promoter and enhancer regions. Enrichment of H3K27ac is also an active chromatin mark [37, 38]. Basically, RNA polymerase II binds to promoter region of genes for transcription initiation with aid of transcription factors. Most mammalian RNA polymerase II initiate transcription at CpG islands, which are devoid of DNA methylation [16]. UCSC analysis confirmed the presence of sites enriched by RNAP II and a CpG island in regulatory region of *RAX* gene. Collectively, ~ 3.2 kb upstream of *RAX* gene was considered as the putative promoter region. The promoter activity of this region was experimentally investigated by using deletion constructs deriving EGFP reporter. Deletion analysis of ~ 3.2 kb human *RAX* promoter regions (− 3097 to the + 167) was analyzed in 293T and ESCs-derived hRPCs. The results of deletion construct analysis showed that region from − 3097 to − 336 (*RAX*-2761) and − 3097 to − 90 (*RAX*-3007) of human *RAX* gene could not independently drive downstream EGFP expression after elimination of minimal regions which basically include general regulatory binding sites required to trigger transcription. These findings indicated that the minimal *RAX* regulatory promoter regions, from − 106 to + 161 (*RAX*-267) and − 364 to + 161 (*RAX*-525), consist of critical elements to derive *RAX* gene expression. Candidate distal region − 3097/+ 161 (*RAX*-3258) and proximal region 1504/+ 161 (*RAX*-1665) showed a significantly higher EGFP expression in both 293T cells and hRPCs. Interestingly, distal region (*RAX*-3258 bp) demonstrated a remarkable more EGFP expression than *RAX*-1665 bp in hRPCs. Based on our results, we speculated that potential elements which are located in distal region, mediate the transcriptional stimulation and contributes to the higher promoter activity of *RAX*-3258 compared to *RAX*-1665.

Previous studies confirmed the necessity of conserved noncoding sequence 1 (CNS1) which is located ~ 2 kb upstream of *Rax* promoter as a regulatory region for expression of *Rax* in mice. CNS1 contains a highly conserved binding sites for Sox2 and Otx2 transcription factors across vertebrates, which are required for *Rax* transcription in mice [18]. These studies identified that Sox2 and Otx2 are potent modulators of *Rax* expression by direct binding to the promoter region and synergistically activate its transcription. Interactions between Sox2 and Otx2 proteins, regulate the expression of *Rax* during eye development [18].

Our bioinformatic analysis using JASPAR, GTRD and UCSC was also predicted binding sites for SOX2 and OTX2 in distal regulatory region of *RAX* gene. This key conserved region was included in *RAX*-3258 bp distal promoter which exhibited the most promoter activity in hRPCs with high expression of SOX2 and OTX2. Interestingly, this candidate region was resulted in substantial reduced expression of EGFP in 293T cells which do not express SOX2 or OTX2 endogenously [18]. Albeit, the roles of other transcription factors in the regulation of human *RAX* expression should not been ignored.

**Fig. 5:**
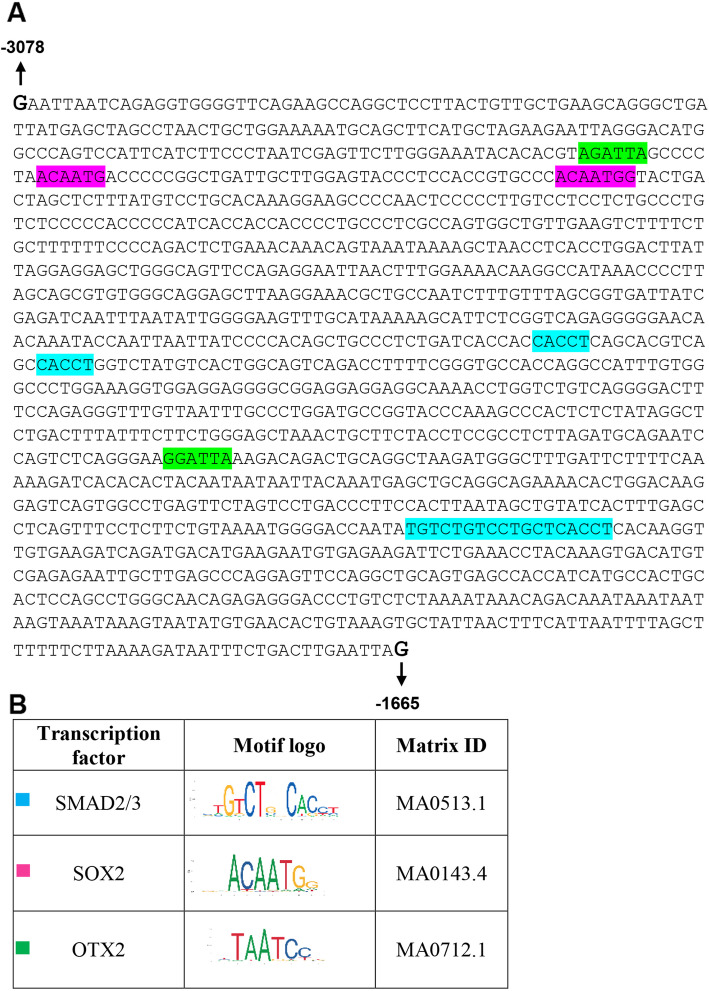
TFBS analysis of human *RAX* distal promoter region (− 1665 to − 3078) (A) Putative binding sites of SOX2, OTX2 and SMAD2/3 within this region were analyzed using the EPD database. (B) Profile summery of transcription factor binding sites using JASPAR data base

Our in silico analysis also revealed several binding sites for SMAD2/3 in distal region of human *RAX* gene. Previous studies of in vitro differentiation of mouse ESCs into retinal cells, identified SMAD2/3 as a key regulator of several retinal genes like *Rax* [39, 40]. Moreover, this study showed that SMAD2/3 was able to directly bind to regulatory elements of retinal and photoreceptor precursor genes. In fact, SMAD2/3 binds to Smad binding elements (SBEs) which are located in distal promoter regions of target genes such as *RAX* and activates their expression (Fig. 5A, B).

For further studies-regarding these molecular mechanisms in human, the effect of corresponding transcription factor overexpression on *RAX* promoter activity with mutated TFBSs can be investigated.

## Conclusion

In summary, the present study introduced the regulatory region of *RAX* gene with high promoter activity. When, this region is included in an expression vector expressing EGFP, it may provide a molecular tool for monitoring retinal progenitor cell maintenance during in vitro culture. Furthermore, our study can be extended towards future investigations regarding the molecular mechanisms by which RAX play key roles in proliferation and development of retinal progenitor cells and reveal more aspects of hRPCs regulation.

## Methods

### Bioinformatics analysis

The sequence of human *RAX* gene was obtained from National Center for Biotechnology Information (NCBI). Different bioinformatic tools were used to predict the potential promoter regions of human *RAX* gene. These software are as follows; UCSC (http://genome.ucsc.edu/), and Genomatix (https://www.genomatix.de/). Also, the putative transcription factor binding sites (TFBS) within the *RAX* promoter region were analyzed using the gene transcription regulation database GTRD (http://gtrd.biouml.org/) and JASPAR (http:// http://jaspar.genereg.net/).

### Primers design for amplification of putative human *RAX* promoter regions

Primers used to amplify the potential promoter regions of the *RAX* gene were designed from National Centre for Biotechnology Information (NCBI) database and confirmed by Primer-BLAST and Oligo7. The primers were reconstituted in nuclease-free water to a concentration of 10 pM/μl. Then, 25 μl PCR reactions containing 2 μl of human genomic DNA (50 ng) as template, 1 μl of each forward and reverse primers (10 pM/μl), 1X Multiplex PCR Master Mix (Yekta Tajhiz Azma Co., Tehran, Iran), and 6 μl of nuclease-free water were prepared, Amplification was carried out in thermocycler (Thermoscientific) with amplification conditions shown in Table 1.

**Table 1:** List of primers for amplification and characterization

Putative Promoter regions /Genes	Primers	Sequences (5′ > 3′)
*OCT4*	Sense Anti-sense	TCTATTTGGGAAGGTATTCAGC ATTGTTGTCAGCTTCCTCCA
*NANOG*	Sense Anti-sense	CAGCTACAAACAGGTGAAGAC TGGTGGTAGGAAGAGTAAAGG
*NESTIN*	Sense Anti-sense	TCCAGGAACGGAAAATCAAG TTCTCTTGTCCCGCAGACTT
*SIX3*	Sense Anti-sense	TCCTCCTCTTCCTTCTCC GTTGTTGATAGTTTGCGGTT
*RAX*	Sense Anti-sense	CAACTGGCTACTGTCTGTC CTTATTCCATCTTTCCCACCT
*PAX6*	Sense Anti-sense	CAGCTCGGTGGTGTCTTTG AGTCGCTACTCTCGGTTTA
*RAX-*267	Sense	ATTAATAGAGAAGGGGCTGGGT
*RAX*-525	Sense	ATTAATTAGTCTGAAGTGAGAGG
*RAX*-1665	Sense Anti-sense	GTCGACGAATTAATCAGAGGTGG AGATCTCTTTGGAGACGGAGAGG
*RAX*-2761	Anti-sense	AGATCTCTCCCCTTGCGTTTGT
*RAX*-3007	Anti-sense	AGATCTACCCAGCCCCTTCTC
*RAX*-3258	Sense Anti-sense	GTCGACGAATTAATCAGAGGTG AGATCTCTTTGGAGACGGAGAGG

### Cloning of putative human *RAX* promoter regions into pEGFP-C1 vector

Using the primers listed in Table 1, the candidate promoter region − 3097/+ 161 of the human *RAX* gene, and a series of control fragments (− 106/+ 161, − 364/+ 161, − 1504/+ 161, − 3097/− 336, − 3097/− 90) were amplified by PCR from human genomic DNA and inserted into *Sal*I/*Bgl*II site of the pEGFP-C1 vector upstream of EGFP reporter. The final expression vector was transformed into *E. coli* DH5-α cells. The integrity of all target sequences was verified by sequencing before evaluation of promoter activity in target cells.

### In vitro cell culture of human embryonic stem cells

RH6 human embryonic stem cells (hESCs, Royan institute) were seeded on 0.3 mg/ml Matrigel (Sigma-Aldrich, St. Louis, MO)-coated tissue culture dishes containing DMEM/F12 medium supplemented with 20% knockout serum replacement (KSR), 0.1 mM nonessential amino acids, 2 mM L-glutamine, 1% ITS and 100 ng/ml bFGF with daily medium exchange (Fig. 1B).

### Differentiation of hESCs into hRPCs

The process of retinal differentiation was briefly demonstrated in Fig. 1A. For neural retinal differentiation, the over confluent feeder-free hESCs were dissociated and then a mechanical approach was used to initiate embryoid body formation (EBs) (Fig. 1C). So hESCs were transferred to low adherent dishes in order to form EBs in neural induction medium (NIM) containing 1 ng/ml noggin (R&D, 1976-NG), 3 μM IWR (R&D, 5439-DK/CF), and 5 ng/mL human recombinant insulin-like growth factor-1 (IGF-1) (R&D, 291-GI) in DMEM/F12 medium supplemented with 10% KSR, 0.1 mM nonessential amino acids, 2 mM L-glutamine, and 1% B27 (Gibco, 17,504–044) for 3 days. In the next step, the EBs were dissociated by Accutase (Millipore, SCR005) and were replated on 1 mg/ml laminin and 15 mg/ml Poly-L-ornithine (both from Sigma-Aldrich) coated 6-well tissue culture plates. The culture medium was replaced with retinal determination medium (RDM) containing DMEM/F12 supplemented with 1% B27, 2% N2 (Gibco, 17,502–048), 10 ng/ml noggin, 3 μM IWR, 10 ng/ml IGF-1, and 10 ng/ml bFGF as previously explained. This medium was exchanged every 2 days up to 21 days (Fig. 1D, E).

### Transient transfection

For investigation the promoter activity of expression vectors harboring the potential promoter regions of *RAX* gene by transfection into 293T cell line, these cells were maintained in DMEM supplemented with 1% L-glutamine, 10% fetal bovine serum, 100 U/ml penicillin and 100 μg/ml streptomycin and incubated at 37 °C in 5% CO2. Next equal moles of expression vectors were transfected into 293T cells [41] by Lipofectamine LTX Transfection Reagent according to the manufacturer’s instructions (Invitrogen, Germany) in a 24-well cell culture plate. Also control groups were transfected by pEGFP-C1 and promoter-less vector (pEGFP-np) for analysis the efficiency of transfection and confirmation of target promoter’s activity respectively. The expression of EGFP reporter resulting from promoter activity of *RAX* regulatory regions were assayed 48 h post transfection. To explore the potential promoter activity of these regions in retinal progenitors, they were also transfected into hESCs- derived hRPCs, as described above.

### Immunocytochemical analysis

To evaluate the expression of stem cell and retinal progenitor markers, immunocytochemistry staining was carried out using the primary antibodies: PAX6 (SC-11357, 1/50), RAX (LS-C53650, 1/200), NESTIN (ab-22,035, 1/100), LHX2 (SC-81311, 1/100) and secondary antibodies Anti-Mouse IgG-FITC (Sigma, AP124F) and Anti-Rabbit IgG-TRITC (Sigma, T6778). Briefly, 5 × 10^4^ cells/well coverslips coated with Matrigel were plated in 24-well plates. One day later, samples were fixed with 4% paraformaldehyde for 30 min at room temperature. Subsequently, these cells were permeabilized by 0.4% Triton X-100 for 30 min and were stained with blocking solution-diluted primary antibodies (BSA, 10 mg/ml) and kept at 4 °C overnight. Then, they were treated with secondary antibodies at 37 °C for 1 h. Furthermore, cell nuclei were stained with DAPI (3 ng/ml, Invitrogen). The images were taken by fluorescent microscope (Olympus, Center Valley, PA, USA) equipped with an Olympus DP70 camera.

### Quantitative PCR (qPCR)

The total RNA of hRPCs and RH6 cells (as negative control) was extracted using RNeasy Plus Mini Kit (Qiagen, Hilden, Germany). Then, cDNA was synthesized using Takara cDNA Synthesis kit using random hexamer primers. All qRT-PCR reactions were performed in triplicate, and data were normalized to human GAPDH mRNA. Relative fold changes in target gene expression was calculated using 2^−ΔΔCt^ method. Table 1, represented primer sequences used in quantitative PCR. Moreover, to investigate the stemness state and multipotency capacity of hESCs-derived hRPCs, the relative expression of several markers including *OCT4*, *NANOG* and eye field markers like *NESTIN*, *SIX3*, *PAX6* and *RAX* were evaluated [11]. Each experiment, had a negative template control (NTC) for primer specificity analysis and lack of DNA contamination. SYBR Green I Master reaction mix (Thermo Fisher Scientific) was used for qPCR analysis of gene expression, and amplification was detected with Light Cycler 480 ABi System.

### Flow cytometry analysis

Flow cytometry assessment was performed to evaluate the quantification of EGFP reporter derived by promoter regions of *RAX* gene. For this purpose, 2 days after transfection of target cells with expression vectors, cells were detached and re-suspended in cold PBS^**−**^ and analyzed by FACS Vantage flow cytometry (Becton Dickinson). Data were analyzed using BD Cell Quest Pro and WinMDI 2.9 software.

### Statistical analysis

All data were analyzed using one-way ANOVA, Tukey’s post-hoc analysis and Student’s t-test and are shown as the mean ± SD. In each experiment, at least three biological replicates were examined. In this study, *P ≤ 0.05* was considered as statistically significant.

## Data Availability

The datasets analysed during the current study are available in the [GenBank] repository, [accession number MZ427311].

## Abbreviations

hRPCs: Human retinal progenitor cells
RAX: Retina and anterior neural fold homeobox
RP: Retinitis pigmentosa
AMD: Age-related macular degeneration
RDD: Retinal degeneration disease
EFTF: Eye field transcription factor
CNS: Conserved noncoding sequence
hESC: Human embryonic stem cell
TFBS: Transcription factor binding site
